# Leadership, collaboration and using data science to fight COVID-19 in the WHO African region

**DOI:** 10.1017/S0950268821000728

**Published:** 2021-04-08

**Authors:** Matshidiso Moeti

**Affiliations:** WHO Regional Office for Africa, Brazzaville, Congo

COVID-19 first arrived in Africa in February 2020, with initial cases introduced by travellers from Europe into Egypt and Algeria [1]. By the end of 2020, there were 1 842 444 cases and 40 958 deaths reported across the 47 Member States within the WHO African region. These represented 2.3% of the confirmed COVID-19 cases and 2.3% of COVID-19-related deaths globally [2]. This figure, although small, is significant in a region that records more than 100 significant public health emergencies annually [3], many of which result in high morbidity, mortality, disability and socioeconomic disruptions, threatening national, regional and global health security. As a result, the region developed robust infectious disease control regimens well before the current COVID-19 pandemic [4–6].

However, the COVID-19 pandemic represented, for the first time, an infectious disease threat that has affected the entire region almost simultaneously, resulting in both public health consequences and major social and economic disruption, with large-scale implications for regional and global health security. The sheer scale of the pandemic has required leadership at the very highest levels, both regionally and nationally. This is predicated on the known and established links between politics, science, security and economy that have been evident in the responses to infectious disease outbreaks and other emergencies across the WHO African region, such as Ebola virus disease outbreak in West Africa in 2014–2016, pandemic influenza (H1N1) in 2009, Ebola virus disease in the Democratic Republic of the Congo between 2017 and 2020 and a multitude of other infectious disease outbreaks during this time, perhaps unparalleled in any other region.

The severity of the 2014–2016 Ebola virus disease outbreak in West Africa galvanised the global community and stimulated global efforts aimed at building capacity for prevention, detection and rapid response to outbreaks and health emergencies. This was in addition to the many years of preparing for and responding to other major regional health issues such as poliomyelitis and HIV/AIDS. The WHO Health Emergencies Programme (WHE) was conceived in this context to support the Member States to rapidly detect and respond to outbreaks and health emergencies. WHE not only complemented WHO's technical and normative role, but has also developed new operational capacities and capabilities for its work in outbreaks and with humanitarian agencies. This was brought about by using an all-hazards approach that brings speed and preparedness to WHO's response to emergencies. It has overseen the rapid assessment of International Health Regulations (IHR) 2005 core capacities. At the same time, road maps have been developed for strengthening public health security in the region, as articulated in the IHR Monitoring and Evaluation Framework.

These initiatives have tangible, measurable outcomes in terms of number of events detected, speed of detection of events and speed of response to events [3]. Time to detection of events decreased from 14 days in 2017, to 7 in 2018 and 4 in 2019, while outbreaks ended faster; the time to declaration of end of outbreak decreased from 131 days in 2017 to 45 days in 2019. Early detection and rapid response to outbreaks greatly contributed to reducing the illness and death associated with these events. Moreover, improved communication and transparency during outbreaks is evidenced by systematic notification of more than 348 substantiated public health events reported to WHO in the African region from 2017 to 2019 [3]. Critical information products, such as the *Weekly Bulletin on Outbreaks and Emergencies* [7], along with regular external situation reports and other *ad hoc* publications, contribute to this.

This supplement to *Epidemiology and Infection* carries a series of papers that highlight the way that the Emergencies Preparedness and Response Programme in the Regional Office for Africa has responded to the challenges of COVID-19 response across this large and diverse region. It has been focusing on data collection and information strategies, and how these data have helped to inform an evidence-based response by countries, along with guidance for risk-based decision making. At the same time, the data collected have been used to provide detailed analysis of the trajectory of the pandemic in the region, as well as a more detailed look at specific metrics, such as morbidity and mortality in selected countries and the effects of non-pharmaceutical interventions on the evolution of the outbreak.

The fight against infectious diseases and other health emergencies in the region requires greater collaboration between health and non-health sectors, encompassing a One Health approach. This has been strengthened regionally by the momentum gained during the assessment of IHR 2005 core capacities and has served as a platform for enhancing the response to the COVID-19 pandemic. Coordination of all efforts to control outbreaks at regional level through the Incident Management Support Team led by the Regional Director for Africa, national task forces in countries led by heads of states, continental coordination at African Union level and the synergy between the WHO Regional Office for Africa and this body has played a central role. In addition, the establishment of a United Nations and African Union joint logistics platform, including the vaccine access programmes developed by the African Union, as well as that of the United Nations and GAVI, has enabled better and more coordinated resource sharing in the African continent.

As the COVID-19 pandemic spread across the African region, countries have moved from preparedness to response. The relatively late introduction of the virus into the region allowed us to learn from the experiences of the rest of the world. The establishment and implementation of the WHO COVID-19 Strategic Response Plan in the WHO African Region [8] gave national authorities the impetus to focus on the pandemic, as well as other priority health areas. As the region continues to experience the effects of the pandemic, we must take the opportunity to expand and intensify collaboration with regional centres of excellence, such as universities and other institutions, as well as international bodies in order to address not only COVID-19, but also ongoing urgent public health priorities.

The past 12 months of pandemic response have highlighted the critical importance of data systems and digital capacity to health system strengthening, along with pandemic data analysis and innovations in technology. This has brought to the fore the importance of continuing funding for strengthening the implementation of the IHR 2005, to maintain and improve health security across the region. The WHO Regional Office for Africa remains available to continue to support the region, working closely with national authorities and partners, to ensure that the lessons learnt during the pandemic continue to be translated into tangible improvements in regional health security.
